# Population Structure and Phylogenetic Relationships in a Diverse Panel of *Brassica rapa* L.

**DOI:** 10.3389/fpls.2017.00321

**Published:** 2017-03-13

**Authors:** Kevin A. Bird, Hong An, Elodie Gazave, Michael A. Gore, J. Chris Pires, Larry D. Robertson, Joanne A. Labate

**Affiliations:** ^1^Division of Biological Sciences, University of MissouriColumbia, MO, USA; ^2^National Key Lab of Crop Genetic Improvement, Huazhong Agricultural UniversityWuhan, China; ^3^Plant Breeding and Genetics Section, School of Integrative Plant Science, Cornell UniversityIthaca, NY, USA; ^4^Plant Genetic Resources Unit, United States Department of Agriculture-Agricultural Research ServiceGeneva, NY, USA

**Keywords:** *Brassica rapa*, genotyping-by-sequencing, population structure, diversity panel, phylogenetic analysis

## Abstract

The crop species *Brassica rapa* L. has significant economic importance around the world. However, the global distribution and complex evolutionary history of the species has made investigating its genetic population structure difficult. Crop domestication and improvement has resulted in extreme phenotypic diversity and subspecies that are used for oilseed, food for human consumption, and fodder for livestock. These subspecies include the oilseed morphotypes. *oleifera* (turnip rape), ssp. *dichotoma* (brown sarson/toria), ssp. *trilocularis* (yellow sarson); ssp. *rapa* (turnip); and Asian leafy vegetables ssp. *pekinensis* (Chinese cabbage), ssp. *chinensis* (bok choy), ssp. *nipposinica* (mizuna/mibuna), ssp. *rapifera* (rapini/broccoli rabe), ssp. *narinosa* (tatsoi), ssp *parachinensis* (choy sum), and ssp. *perviridis* (komatsuna). To date, studies have had insufficient sampling to determine the relationship of all morphotypes, especially oilseed morphotypes, and questions remain over the contribution of morphotype and geographic origin to population structure. We used genotyping-by-sequencing to score 18,272 single nucleotide polymorphism markers in a globally diverse panel of 333 *B. rapa* National Plant Germplasm System accessions that included 10 recognized subspecies. Our population genetic and phylogenetic analyses were broadly congruent and revealed five subpopulations that were largely reflective of morphotype and geography. These subpopulations were 1. European turnips/oilseed, 2. Asian turnips/oilseed, 3. yellow/brown sarson (ssp. *trilocularis* and ssp. *dichotoma*), 4. Chinese cabbage (ssp. *pekinensis*), and 5. bok choy, choy sum, and tatsoi (ssp. *chinensis*, ssp. *parachinensis*, ssp. *narinosa*). Additionally, we found evidence of polyphyly and/or paraphyly, particularly for oilseed morphotypes (ssp. *oleifera* and ssp. *dichotoma*) and turnips. The results of this study have provided improved resolution to the genetic and phylogenetic relationships of subspecies within the species *B. rapa*. Understanding of these relationships is key to the future genetic study and improvement of this globally important crop species.

## Introduction

*Brassica rapa* L. (genome AA, 2*n* = 2*x* = 20) is an agriculturally important food crop, consisting of morphotypes that produce leafy vegetables, swollen root vegetables, and vegetable oil (Cartea et al., 2011). These subspecies include the oilseed morphotypes ssp. *oleifera* (turnip rape), ssp. *dichotoma* (brown sarson/toria), ssp. *trilocularis* (yellow sarson); ssp. *rapa* (turnip); and Asian leafy vegetables ssp. *pekinensis* (Chinese cabbage), ssp. *chinensis* (bok choy), ssp. *nipposinica* (mizuna/mibuna), ssp. *rapifera* (rapini/broccoli rabe), ssp. *narinosa* (tatsoi), ssp. *parachinensis* (choy sum), and ssp. *perviridis* (komatsuna). These various subspecies have traditionally been classified by crop use and morphology (Bonnema et al., 2011; McGrath and Quiros, 1992).

This system of nomenclature has led to a complicated history of *Brassica* taxonomy. While generally the Asian leafy vegetable and turnip groups are well-defined and stable, turnip-rape and oilseed rape have been debated among systematists since the mid-nineteenth century (Gupta and Pratap, 2007). For example, Hooker and Thompson (1861) considered the oilseed varieties yellow sarson and toria/brown sarson with two valved fruit as one species, while the three and four valved varieties were considered to be their own respective species. Alternatively, Duthie and Fuller (1882) considered yellow sarson a variety of *B. rapa* and brown sarson and toria a variety of *Brassica napus* L. (genome AACC, 2*n* = 4*x* = 38). After Olsson (1954) suggested that all *Brassica* species with 20 chromosomes be grouped into *Brassica campestris* (syn. *rapa*), Singh (1958) argued yellow and brown sarson were varieties of *B. campestris* (syn. *rapa*), while Prakash (1973) claimed they were both forms of ssp. *oleifera*. The currently accepted nomenclature considers yellow sarson to be ssp. *trilocularis* and brown sarson and toria to be ssp. *dichotoma* (Hanelt and Büttner, 2001). Beyond the difficulties of taxonomically grouping numerous intraspecific varieties, the failure to recognize distinctions among parent lines can sometimes lead to seeds of different species being mixed together in germplasm collections, which further complicates taxonomic assignment of *Brassica* crops (Gupta and Pratap, 2007).

Previous population genetic and phylogenetic studies aimed at disentangling the relationships among subspecies of *B. rapa* have used an array of molecular marker types including isozyme, restriction fragment length polymorphism (RFLP), random amplified polymorphic DNA (RAPD), amplified fragment length polymorphism (AFLP), and simple sequence repeat (SSR) (McGrath and Quiros, 1992; Das et al., 1999; Zhao et al., 2005, 2007, 2010; Takuno et al., 2007; Del Carpio et al., 2011a,b; Guo et al., 2014). More recent studies have capitalized on whole-genome sequencing and single nucleotide polymorphism (SNP) genotyping approaches such as amplicon sequencing (AmpSeq) and genotyping-by-sequencing (GBS) (Cheng et al., 2016a,b; Tanhuanpää et al., 2016).

This existing body of work has two major shortcomings. The first is the lack of consensus on whether genetic structure and population grouping are reflective of geographic origin or morphotype. The second is insufficient sampling in previous studies to estimate the phylogenetic relationship of major *B. rapa* subspecies, especially with regards to oilseed types like ssp. *dichotoma* and ssp. *trilocularis*. For example, Guo et al. (2014) found three main groups related to geographic origin (Europe/North Africa, East Asia, and “mixed”) from genotyping 51 SSRs in 173 accessions. However, using AFLP markers other studies used 160 accessions and found four groups (Zhao et al., 2007; Del Carpio et al., 2011a) or used 239 accessions and found five groups (Zhao et al., 2010) that reflected broad morphotype varieties. Recently, using 209 SNPs, Tanhuanpää et al. (2016) identified three main groups related to morphotype and flowering habit of 61 accessions. Furthermore, the populations used in the studies of Guo et al. (2014), Zhao et al. (2005, 2007, 2010), Del Carpio et al. (2011a,b), Dixon (2007), Tanhuanpää et al. (2016), and Cheng et al. (2016a,b) suffer from little (one to two accessions) or no representation of subspecies such as ssp. *dichotoma* and ssp. *trilocularis*. When performing estimations of population structure and ancestry, under sampling and sampling bias can produce large error rates if not accompanied by a large number of loci (Shringarpure and Xing, 2014). The inconsistency of the results of previous studies also potentially stems from shortcomings in the types of molecular marker data as well as populations used. The use of these AFLPs is plagued by problems of homoplasy and dominant inheritance (Schlötterer, 2004; Mba and Tohme, 2005; Frascaroli et al., 2013) while SSRs suffer from homoplasy, sequences sometimes being associated with multiple SSR lengths, and complex mutational patterns (Schlötterer, 2004; Frascaroli et al., 2013). This further highlights the importance of using modern sequencing technology to generate whole genome and genome-wide SNP data.

Recently, *B. rapa* researchers have been able to harness the advances made in DNA sequencing technology. Cheng et al. (2016a,b) used a random 20,000 SNP subset of 2.2 million SNPs called from whole-genome sequence data of 199 accessions of *B. rapa* and reported six STRUCTURE groups broadly corresponding to morphotypes (1. European turnip; 2. Chinese cabbage; 3. ssp. *parachinensis* (choy sum); 4. ssp. *chinensis var. purpurea* (Zicatai), 5. bok choy (ssp. *chinensis*), 6. A group found to equally represent Japanese accessions, ssp. *oleifera*, ssp. *trilocularis*, Chinese turnips, and some admixed European turnips). However, when phylogenetic analyses based on 6,000 SNPs shared with *B. oleracea* (genome CC, 2*n* = 2*x* = 18) were performed there were 7 groups presented (1. European turnips, 2. sarson and rapid cycling, 3. Chinese turnip, 4. turnip rape (ssp *oleifera*), 5. Japanese turnips and mizuna (ssp. *nipposinica*), 6. bok choy (ssp. *chinensis*) and related leafy green morphotypes, and 7. Chinese cabbage (ssp. *pekinensis*). Accessions belonging to Asian turnips and oilseed groups (ssp. *oleifera* and ssp. *trilocularis*) that were indicated as monophyletic clades in phylogenetic analysis showed large amounts of admixture from STRUCTURE. Placement of ssp. *oleifera* was also observed to be discordant between the two methods.

Other innovative SNP discovery strategies based on the construction of highly multiplexed, reduced representation libraries (RRLs) enable the production of massive SNP data sets for very large populations at low cost per sample compared to whole-genome sequencing (Altshuler et al., 2000; Davey et al., 2011; Poland and Rife, 2012). Another benefit of high-throughput DNA sequencing methods such as GBS (Elshire et al., 2011) is that ascertainment bias can be reduced by using pipelines that allow marker discovery and genotyping to occur simultaneously (Poland and Rife, 2012).

In this study, we investigated the population structure and phylogenetic relationships of *B. rapa* subspecies using a high-throughput GBS method that leverages next-generation sequencing and multiplexing of RRLs to discover and score tens-of-thousands of SNPs across hundreds of *B. rapa* samples. Our improved population sampling, particularly regarding oilseed accessions such as ssp. *dichotoma* and ssp. *trilocularis*, provided an opportunity to improve on findings of previous studies and support novel insights into how *B. rapa* subspecies relate to each other.

## Materials and methods

### Germplasm

**Figure 1:**
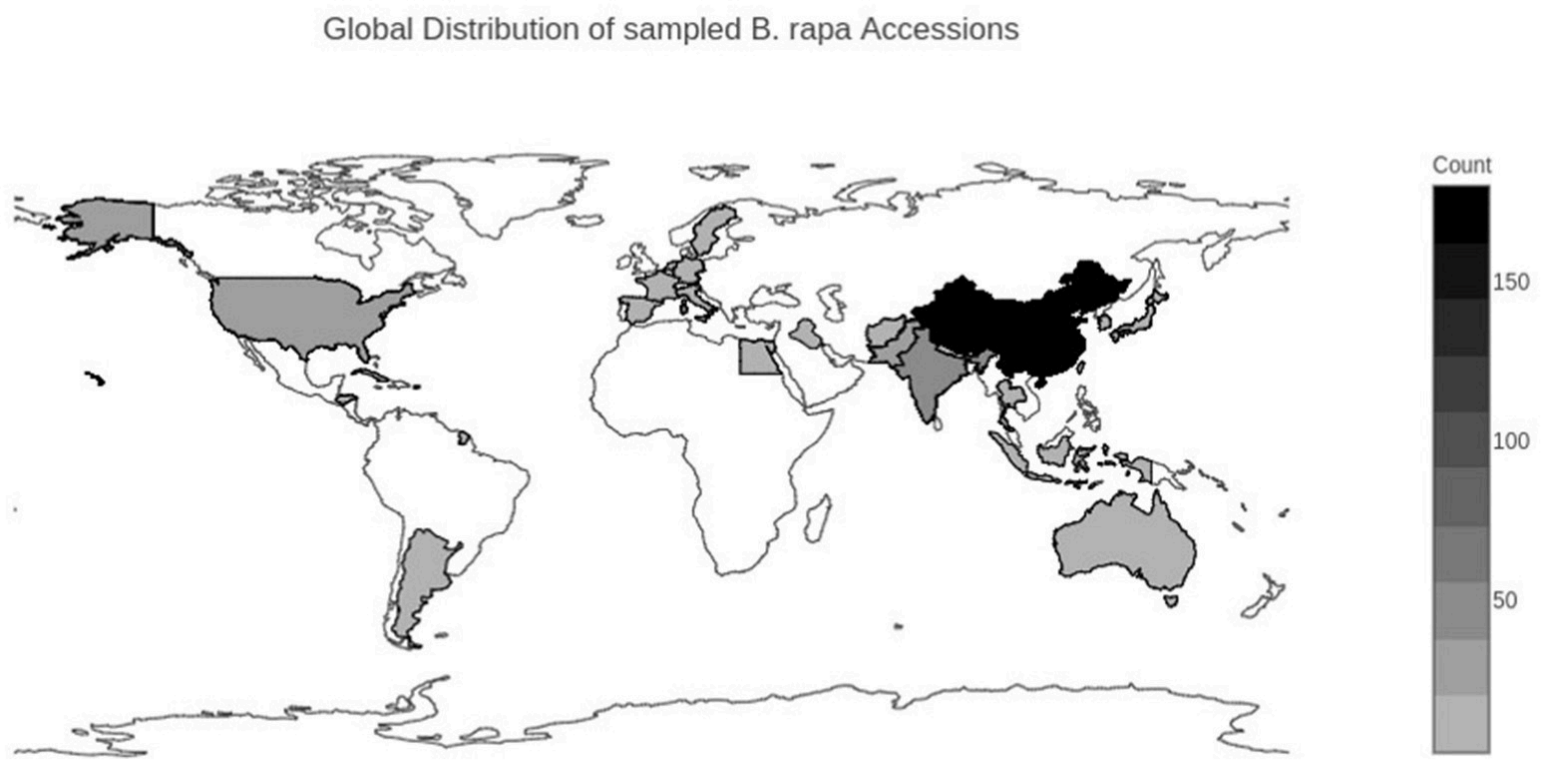
**Geographical distribution of sampled *Brassica rapa* L. accessions**. Darker colors represent a larger number of accessions from that country. Country of origin was determined from the USDA GRIN-Global database.

We selected 364 accessions from the USDA National Plant Germplasm System (NPGS) (Supplementary Table 1). Of the 364 accessions, 264 were from the USDA Plant Genetic Resources Unit (NE-9) in Geneva, NY. These 264 accessions were composed of 149 accessions of Chinese cabbage (ssp. *pekinensis*), 65 accessions of bok choy (ssp. *chinensis*), 17 accessions of turnip (ssp. *rapa*), 4 accessions of mizuna (ssp. *nipposinica*), 4 accessions of oilseed rape (ssp. *oleifera*), 2 accessions of tatsoi (ssp. *narinosa*), 2 accessions of choy sum (ssp. *parachinensis*), 2 accessions of komatsuna (ssp. *perviridis*), 1 accession of brown sarson/toria (ssp. *dichotoma*), and 18 accessions without an identified subspecies. The NE-9 germplasm represented the total available vegetable collection of *B. rapa* for NPGS. An additional 100 accessions were selected from the ~600 total accessions from the USDA North Central Regional PI Station (NC-7) in Ames, IA. These 100 accessions were composed of 21 accessions of yellow sarson (ssp. *trilocularis*), 9 accessions of brown sarson/toria (ssp. *dichotoma*), 4 accessions of oilseed rape (ssp. *oleifera*), 2 accessions of choy sum (ssp. *parachinensis*), 1 accession of turnip (ssp. *rapa*) and 63 accessions with no identified subspecies. Accessions from NC-7 were chosen to maximize geographic and subspecies diversity. Geographic origin was determined according to the listed country of origin reported in the USDA NPGS GRIN-Global Passport data (Figure 1) (Supplementary Table 1). Country level data were qualitatively grouped into broad geographic ranges of the Americas, Europe, East Asia, and South/Central Asia/Middle East, and Australia. The final population analyzed was composed of 333 of the original 364 accessions (see taxon quality filtering section).

### DNA isolation and genotyping-by-sequencing

The 364 accessions were grown in a greenhouse at the University of Missouri-Columbia. Fresh leaf tissue was harvested from the youngest true leaf (leaf 3–4) 4 weeks after planting. Leaf tissue samples were stored at −80°C until DNA was isolated. Total genomic DNA isolation was conducted using the DNeasy 96 plant kit (Qiagen, USA) with minor modifications of the protocol (Labate and Robertson, 2015). DNA concentration was quantified using PicoGreen reagent (Thermo Fisher Scientific, Waltham, MA) and a TECAN Infinite 200 PRO (Tecan US, Morrisville, NC) microplate reader. Samples that had less than 1 μg of DNA at 100 ng/μL, were heavily degraded (fragments <20 kb), or failed to be completely digested by the restriction enzyme EcoRI were discarded and replaced according to the above extraction protocol. For the test restriction enzyme digestion, only a random 10% of samples were tested with EcoRI.

GBS libraries were constructed using the restriction enzyme ApeKI and the standard barcode and common adapter sets (Elshire et al., 2011). Sequencing of single end 86 bp reads of four 96-plex GBS libraries was conducted at the Cornell University Genomics Diversity Facility with a Genome Analyzer II (Illumina, Inc, San Diego, CA) using one flowcell lane per library. GBS output files were analyzed using the GBS pipeline v1 from TASSEL4 using default settings (Glaubitz et al., 2014). In brief, raw GBS reads with at least five copies were sorted into tags (aligned short reads). The tags were then aligned to the *B. rapa* reference genome var. Chiifu 1.29 (Wang et al., 2011) using the Burrows-Wheeler Aligner (BWA) v. 0.7.13 program on default settings (Li and Durbin, 2009). The TASSEL SNP discovery plugin parameters allowed for a maximum mismatch rate of two, called heterozygous sites and ignored indels and sites with more than two alleles (Supplementary File 1). All raw data are available through the National Center for Biotechnology Information (NCBI) Sequence Read Archive (SRA) (BioProject PRJNA374457 and BioSample numbers SAMN06323355–SAMN06323736).

### SNP quality filtering

SNPs were output in the variant call format (VCF) and all low-quality genotypes (GQ <98) were removed using vcftools (Danecek et al., 2011). In order to reduce the probability of paralogous SNPs being called as heterozygous, additional filtering was applied. First, to eliminate high-copy number sites, we filtered out sites with above average site read depth. Mean site read depth reported from vcftools and observed heterozygosity reported from TASSEL4 were plotted together and a mean site read depth of 140 was determined to be a point where site heterozygosity exceeded a threshold of 70% heterozygosity, so sites with a mean read depth greater than 140 were removed. Second, when three or more SNPs were located within 5 bp from each other, these SNPs were removed. This filtering removed both SNPs called from misalignment due to short indel polymorphisms between true alleles and misalignment of paralogs. Third, any site with more than 50% observed heterozygosity was removed. Finally, we removed one DNA sample that had less than 10% of SNP sites scored. These parameters resulted in 18,272 SNPs identified in 363 accessions (Supplementary File 1).

### Taxon quality filtering

To serve as a quality check for genotyping, 17 plants were sampled twice as technical replicates for GBS. Before removing duplicated samples for downstream analyses, all samples were used to generate a Neighbor-Joining tree in PHYLIP v. 3.6 (Felsenstein, 1989) as in Labate and Robertson (2015). All duplicated samples clustered sister to each other in the Neighbor-Joining tree with bootstrap values ranging from 87 to 100%.

Nuclear genome size data based on flow cytometry of fresh leaf tissue from a subset of these lines also indicated that some of the accessions had C-values inconsistent with *B. rapa* and were potentially *B. napus*, autopolyploid *B. rapa*, or another *Brassica* polyploid species (Supplementary Table 2).

In addition, when FastStructure (Raj et al., 2014) was run (see below), the K = 2 results gave a large “*B. rapa*” group and a second group of these “polyploid accessions.” Accessions for which there were no flow cytometry data but shared greater than 10% probability assignment with the “polyploid” subpopulation, were excluded from further analyses along with the “polyploid accessions.”

The remaining samples were analyzed using FastStructure and RAxML (Stamatakis, 2014). Clusters and clades were named as suggested by the identified subspecies of the plurality of samples in each group. The 40 accessions whose USDA GRIN subspecies designation did not match their FastStructure grouping were not removed from the analyses. These potentially misidentified samples represented less than 25% of their respective FastStructure clusters and did not change the conclusions drawn from the data. The total number of accessions after all taxon filtering steps was 333.

### Phylogenetic and population structure

To observe the phylogenetic relationships among the 333 accessions, a maximum likelihood phylogeny was constructed with RAxML version 8.2.4 (Stamatakis, 2014) on the Cipres Science Gateway (Miller et al., 2010) with rapid bootstrapping under the GTR+GAMMA model. The phylogeny was based on the 18,272 SNP data set and the tree was rooted using four accessions (PI 209020, PI 633153, PI 198062, PI 633169) identified as *Brassica* polyploid individuals by flow cytometry (Supplementary Table 2).

Population groups were inferred using the model-based, Bayesian clustering software FastStructure v 1.0 (Raj et al., 2014). FastStructure was run on default settings with 10-fold cross-validation on the 333 accessions testing for subpopulations (K) ranging from K = 2 to 12. The python script ChooseK, included with the FastStructure package, was used to choose the number of subpopulations that maximize the marginal likelihood. Results were graphically represented using STRUCTUREPLOT v1 (Ramasamy et al., 2014) with PI numbers included as individual labels and the Structure plot ordered by *Q*-value (Supplementary Figure 2). Principle component analysis (PCA) was done using the PCA function in the TASSEL 5.2.33 GUI and visualized using the R package ggplot2.

### Genetic diversity

For genetic diversity analyses, subpopulations were defined as the clusters produced by FastStructure at K = 5. The program Arlequin v 3.5 (Excoffier and Lischer, 2010) was used both to calculate pairwise genetic distance (F_ST_) for the subpopulations according Weir and Cockerham (1984) and to compare molecular diversity between and within morphotypes (populations) and geographic regional groups using Analysis of Molecular Variance (AMOVA) (Weir and Cockerham, 1984; Excoffier et al., 1992; Weir and Cockerham, 1996). F_ST_ results were interpreted using the same standard as Del Carpio et al. (2011a), where an F_ST_ of 0 indicates no differentiation between subpopulations and a value of 1 indicates complete differentiation. Populations were considered to have little differentiation when F_ST_ values were less than 0.05, moderate differentiation when F_ST_values were between 0.05 and 0.15, strong differentiation when F_ST_ values were between 0.15 and 0.25, and very strong differentiation when F_ST_ values were greater than 0.25 (Hartl, 1980; Mohammadi and Prasanna, 2003). Finally, the genotype summary module in the TASSEL4 GUI was used to determine observed heterozygosity rate and the proportion of data missing per accession for all genotyped accessions (Supplementary Table 1) (Bradbury et al., 2007).

## Results

### GBS sequencing

We used GBS to analyze a panel of 333 *B. rapa* accessions, which included 10 subspecies. The original 823,954,356 raw reads were filtered to 3,02,5369 tags, of which 42% aligned to the *Brassica rapa* reference genome. Additional sequencing information can be found in Supplementary File 1. The final, filtered data set of 18,272 high-quality SNP markers had an average site depth of 28 reads. The sequenced taxa had an average site depth of 28, were missing 0.6% of SNPs on average, and had an average heterozygosity rate of 10.3%. Of the 18,272 SNPs, 83.6% (15,277) were in exons, 5.1% (921) were in introns, 0.5% (98) were in repetitive regions and 10.8% (1976) were in intergenic regions This bias toward genic and coding regions is expected with RRL techniques like GBS (Elshire et al., 2011). Additionally, previous genomic analysis of *B. rapa* showed a similar pattern of CDS SNP bias over intron and intergenic SNPs (Cheng et al., 2016a,b). However, it was observed that the SNP filtering employed exacerbated this bias, which may represent a departure from the true biological pattern of genomic feature distribution.

### Population structure and phylogenetic analyses

**Figure 2:**
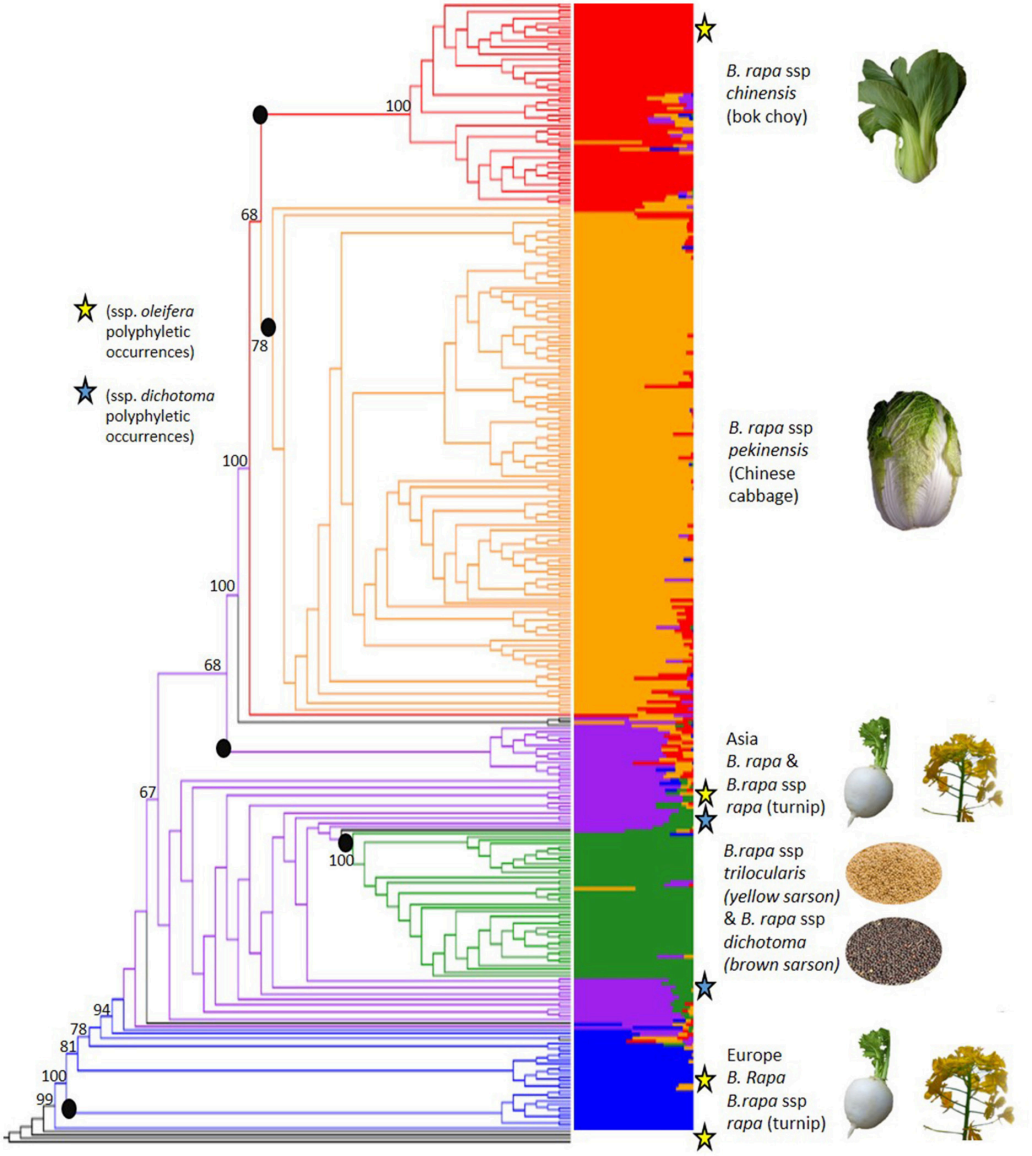
**Results of FastStructure (right**, also see Supplementary Figure 2) and phylogenetic tree from RAxML (**left**, also see Supplementary Figure 3) results based on 18,272 SNPs that were scored on 333 *Brassica rapa* L. accessions. Colors define subpopulations: blue (European turnip/ European oilseed), purple (Asian turnip, South Asian oilseed), green (yellow sarson/brown sarson), orange (Chinese cabbage), and red (bok choy, tatsoi, choy sum). The roots of RAxML monophyletic clades concordant with FastStructure groups are marked with black dots are not numbered as presented in the results. Polyphyletic occurrences of ssp. *dichotoma* are marked with blue stars. Polyphyletic occurrence of ssp. *oleifera* are marked with yellow stars. Admixed accessions with no consensus subpopulation are in black. The phylogeny was rooted with four lines from the panel that were determined to be *Brassica* polyploids and bootstrap values for key nodes are shown on the figure. (See Supplementary File 2 for photo credit and copyright information).

The 18,272 SNPs were used to evaluate the genome-wide patterns of population structure in this globally representative sampling of geographical and morphological diversity in *B*. *rapa*. FastStructure determined that five subpopulations (K = 5) was the optimal clustering for the 333 accessions based on the 18,272 high quality SNP markers (Figure 2, Supplementary Figures 1, 2). The five inferred subpopulations and the number of accessions within were as follows: Subpopulation 1: European turnip/oilseed 28 accessions, subpopulation 2: Asian turnips/oilseed 44 accessions, subpopulation 3: Yellow/brown sarson (ssp. *trilocularis* and ssp. *dichotoma*, respectively) 42 accessions, subpopulation 4: Chinese cabbage (ssp. *pekinensis*) 150 accessions, and subpopulation 5: bok choy (ssp. *chinensis*) 62 accessions. There were an additional seven admixed accessions with no consensus (>50%) population assignment which were not clustered in the above mentioned groups. Overall, 278 of 333 accessions could be confidently placed within these five subpopulations by both FastStructure and RAxML.

Subpopulation 1 was the smallest group (28) and contained nine ssp. *rapa* (turnip) accessions predominantly from Europe and the US. Many of the accessions (17) in this population had no designated subspecies. The GRIN-Global subspecies identifications and the assigned FastStructure populations can be found in Supplementary Table 1. Additionally, the PI numbers of the accessions removed based on flow cytometry can be found in Supplementary Table 2 to aid in curating the NPGS accessions.

Subpopulation 2 consisted of 44 accessions, nine of which were *B. rapa* ssp. *rapa* (turnip) from Japan and the US, four were *B. rapa* ssp. *nipposinica* (mizuna) accessions from Japan and a number of accessions (17) with no identified subspecies from the Middle East/South Asia, some of which were observed to have the swollen hypocotyl turnip phenotype. Passport data from GRIN-Global indicate that the turnip lines from the US in this population were all from the US division of the Japanese seed company Takii, implying they were actually of Japanese origin. This population also contained four *B. rapa* ssp. *dichotoma* accessions, and five accessions with no designated subspecies that were identified in the GRIN-Global database as toria, a variety of *dichotoma*. All nine lines showed contribution from subpopulation 4, suggesting either hybridization or introgression from the yellow sarson subpopulation or shared ancestry.

Subpopulation 3 had 42 accessions total, most were ssp. *trilocularis* (yellow sarson) accessions (20), but there were also three accessions of *B*. *rapa* ssp. *dichotoma* (brown sarson/toria). Within this subpopulation, 10 accessions without subspecies identification were found to have 100% population assignment to subpopulation 3 and yellow seed, and as such inferred to be yellow sarson.

Subpopulation 4 was the largest observed subpopulation with 150 accessions and contained 122 accessions of *B*. *rapa* ssp. *pekinensis* (Chinese cabbage). This subpopulation also contained 23 accessions labeled as *B*. *rapa* ssp. *chinensis* (bok choy). Due to the small number of *chinensis* and the lack of contribution from the *chinensis* FastStructure subpopulation 5 it is possible that the accessions were misidentified in GRIN-Global.

Subpopulation 5 was composed of 62 accessions, mainly *B. rapa* ssp. *chinensis*, but also contained *B. rapa* ssp. *narinosa*, and *B. rapa* ssp. *parachinensis*. The latter is possibly derived from *B. rapa* ssp. *chinensis* (Dixon, 2007). Additionally, 10 ssp. *pekinensis* accessions were part of subpopulation 3.

To confirm membership of subpopulations derived from FastStructure and to further resolve the membership of highly admixed accessions, we used a phylogenetic method based on maximum likelihood. The phylogenetic relationships among accessions were generally well-supported with roughly two-thirds of the nodes having a bootstrap value over 50%. In agreement with the FastStructure analysis, the phylogenetic results revealed the presence of five main monophyletic clades, which showed bootstrap support over 50% and were highly concordant with the FastStructure results (Figure 2, Supplementary Figure 3): Clade 1: European turnips, which contained 11/28 accessions from FastStructure subpopulation 1, clade 2: Asian turnips and mizuna which contained 15/44 accessions from FastStructure subpopulation 2, clade 3: Yellow and brown sarson which contained 42/42 accessions from subpopulation 3, clade 4: Chinese cabbage which contained 150/150 accessions from subpopulation 4, and clade 5. bok choy, tatsoi, and choy sum (ssp. *chinensis, narinosa*, and *parachinensis*, respectively) which contained 61/62 accessions from FastStructure subpopulation 5.

**Figure 3:**
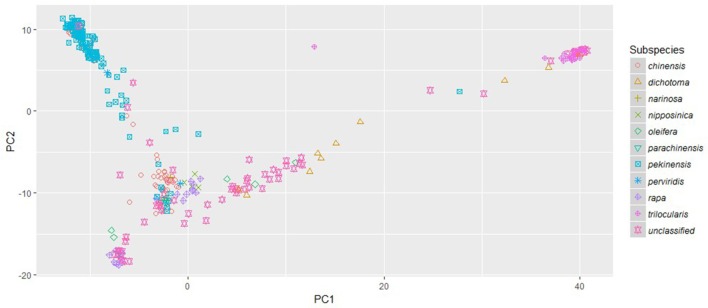
**Principle Component Analysis plot of PC1 and PC2 based on 18,272 genome-wide SNPs scored on 333 *Brassica rapa* L. accessions**. Subspecies, as identified in USDA GRIN-Global, are indicated by colored symbols.

The phylogenetic clusters helped to disentangle the grouping of accessions showing large amounts of admixture. Many of the admixed brown sarson, toria, and South Asian/Middle Eastern oilseed accessions that clustered with Asian turnips in FastStructure subpopulation 2 did not form a well-supported clade, but all were located closer to yellow sarson (clade 3 in RAxML) than any other group, which better follows morphology and domestication history (Gomez Campo and Prakash, 1999). Additionally, a number of oilseed rape accessions and Italian accessions in FastStructure subpopulation 1 were found between the European turnip clade (clade 1 in RAxML) and the unstructured clade of South/Central Asian oilseed accessions that were placed in FastStructure subpopulation 2. These results were reinforced by the PCA which indicated that the South Asian/Middle Eastern accessions including toria and brown sarson were found to group between ssp. *trilocularis* and the rest of the sampled accessions, and the ssp. *dichotoma* accessions were the closest to the ssp. *trilocularis* accessions. Additionally, the above mentioned Italian accessions were located near the European turnip subpopulation 1, but not in the center of the cluster (Figure 3).

### F_ST_ and AMOVA

The genome-wide data set of 18,272 SNP markers was used to estimate the between and within population differentiation for the 333 accessions of *B. rapa*. F_ST_ values between FastStructure subpopulations ranged from moderate between Asian turnip/oilseed vs. bok choy (0.13) and European turnip/oilseed vs. Asian turnip/oilseed (0.15), to very strong (0.66) between yellow sarson vs. European turnip/oilseed (Table 1). AMOVA results indicated that 12.15% of variation was among geographic regional groups, while 11.21% of variation was among morphotypes (populations) within geographic regional groups. Of the remaining variation, 32.69% was among individuals within populations, and 42.95% of variation was within individuals. (Table 2). Due to the observed polyphyly of ssp. *dichotoma*, another pairwise F_ST_ comparison was conducted to measure differentiation between yellow sarson, the ssp. *dichotoma* accessions that grouped with yellow sarson, the accessions identified as toria in GRIN-global, and the ssp. *dichotoma* accessions that clustered with the Japanese turnips and South Asian oilseeds. The pairwise F_ST_ revealed moderate (0.08) differentiation between yellow sarson and the ssp. *dichotoma* that grouped with yellow sarson, as well as little differentiation between toria accessions and the ssp. *dichotoma* that grouped with Asian turnips/oilseed (0.04). F_ST_ values for the remaining combinations showed very strong differentiation (>0.25 Table 3).

**Table 1:** **Pairwise F_ST_ values between the five subpopulations derived from Fast Structure**.

**Asian turnip/oilseed**				**Chinese cabbage**			**bok choy**		**yellow sarson**
**Chinese cabbage**	**bok choy**	**yellow sarson**	**European turnip**	**bok choy**	**yellow sarson**	**European turnip**	**yellow sarson**	**European turnip**	**European turnip**
0.20	0.13	0.45	0.15	0.18	0.57	0.26	0.56	0.21	0.66

**Table 2:** **Analysis of Molecular Variance (AMOVA) results for global F_ST_ statistics**.

**Source of variation**	**d.f**.	**Sum of squares**	**Variance components**	**Percentage of variation**
Among groups	4	67511.06	119.76 Va	12.15
Among populations within groups	20	65484.25	110.50 Vb	11.21
Among individuals within populations	311	335156.65	322.25 Vc	32.69
Within individuals	336	145544.50	433.17 Vd	43.95
Total	671	613696.46	985.68	
Fixation indices	F_SC_: 0.13	F_CT_: 0.12	F_IS_: 0.43	F_IT_: 0.56
Significance tests (1023 permutations)	Vc and F_SC_: *P* <0.00001	Va and F_CT_: *P* = 0.006	Vc and F_IS_: *P* <0.00001	Vd and F_IT_: *P* <0.00001

**Table 3:** **Pairwise F_ST_ values between ssp. *dichotoma* accessions and the FastStructure groups in which they clustered**.

**ssp. *dichotoma* (Asian turnip/oilseed group)**			**ssp. *dichotoma* (yellow sarson group)**		**yellow sarson group**
**ssp. *dichotoma* (yellow sarson group)**	**yellow sarson group**	**toria group**	**yellow sarson group**	**toria group**	**toria group**
0.28	0.52	0.04	0.08	0.33	0.57

## Discussion

The striking phenotypic diversity of *B. rapa* has made it a valuable multi-use crop for food, fodder, and oil. Although the complex domestication history and strong selective breeding of *B. rapa* were instrumental in generating and shaping this diversity, they greatly complicate the elucidation of population structure and evolutionary relationships in the species. By leveraging a large SNP data set in the largest *B. rapa* diversity panel to date (333 samples compared to 239 in the next largest panel), our study provides a more detailed analysis of the relationships of *B*. *rapa* subspecies. With an improved sampling of subspecies, we were able to uncover a complex relationship between ssp. *trilocularis* and ssp. *dichotoma*. Furthermore, unlike some previous studies that emphasized geography (Zhao et al., 2005; Guo et al., 2014) or morphotype (Zhao et al., 2007, 2010; Del Carpio et al., 2011a) as a primary contributor for population clusters, our FastStructure population clustering and RAxML phylogenetic results indicate five subpopulations that predominantly correspond to broad morphological groups such as oilseed, turnip, bok choy, and Chinese cabbage, but show moderate population differentiation between turnips from Japan and Europe. That these broad morphological groups are strongly tied to geography could potentially explain how previous studies derived STRUCTURE subpopulations based on geographic origin. Leafy vegetables such as Chinese cabbage and bok choy originate from East Asia, while sarsons and other oilseed varieties are predominantly from India and Central/South Asia.

### Polyphyly and paraphyly of oilseed *Brassica rapa* accessions

We found evidence for polyphyly in our sampled oilseed accessions (ssp. *oleifera*, and ssp. *dichotoma*). Polyphyly of *B. rapa* ssp. *dichotoma* may be related to the genetic differentiation between toria and brown sarson. Of the eight ssp. *dichotoma* accessions, three clustered in the yellow sarson subpopulation 3 from FastStructure and were within the yellow sarson clade in the RAxML phylogeny. Of the remaining, four clustered with the Asian turnip/oilseed FastStructure subpopulation 2, showing evidence of admixture and over 10% probability assignment to the yellow sarson subpopulation 3, and one was admixed with nearly equal contributions from the yellow sarson and the Asian turnip/oilseed subpopulations (Figure 2). These latter five accessions were closer to the yellow sarson clade in the phylogenetic tree than to any other clade representing a major morphotype group.

For two of the three ssp. *dichotoma* accessions (PI 347594 and PI 349602) that clustered with the yellow sarson subpopulation 3, we observed a mixture of brown and yellow seeds. Yellow and brown seed color in oilseed *B. rapa* is believed to be controlled by digenic inheritance having dominant epistatic interaction, with brown and mixed seed color dominant to yellow seed color (Rahman, 2014). Based on the Guo et al. (2014) report of a number of accessions in their panel with mixed colored seed that were not restricted to a particular subspecies and did not group consistently in their STRUCTURE analysis, it does not seem appropriate to call these accessions misidentified, as seed color appears to naturally segregate, even in non-yellow sarson species.

The remaining five ssp. *dichotoma* accessions grouped closely to five Pakistani accessions from NC-7 with no designated subspecies, but recorded as toria from the breeder's information in GRIN-Global passport data. Of the five ssp. *dichotoma* accessions from FastStructure subpopulation 2, two were directly sister to toria accessions, indicating they may be toria. The genetic identity of the other accessions of *B. rapa* ssp. *dichotoma* could not be further determined.

Pairwise F_ST_ indicated that there were significant genetic differences between the two groups of ssp. *dichotoma* (Table 3). Our results suggest that yellow sarson and some ssp. *dichotoma* accessions are genetically very similar and could be considered a single subspecies, while the other ssp. *dichotoma* accessions and toria are nearly genetically indistinguishable and are potentially another subspecies.

Previous studies using UPGMA clustering based on AFLP, RFLP, SSR, and SNP data failed to sample both ssp. *trilocularis* and ssp. *dichotoma* (Zhao et al., 2005; Takuno et al., 2007; Del Carpio et al., 2011a; Cheng et al., 2016a,b) or found the two subspecies to be distinct monophyletic clades (Das et al., 1999; Rahman et al., 2004; Zamani-Nour et al., 2012). While both Das et al. (1999) and Zamani-Nour et al. (2012) only sampled one accession of brown sarson and yellow sarson, their inclusion of both helped shed light on the phylogenetic relationship of the subspecies. Furthermore, when 23 accessions of brown sarson and 3 accessions of toria were included in Rahman et al. (2004) it was found they formed distinct clades. Additionally, McGrath and Quiros (1992) observed that toria and brown sarson had different levels of heterozygosity, but did not include all three oilseed morphotypes (yellow sarson, toria, and brown sarson) in their phylogenetic analysis. Unfortunately, Das et al. (1999); Rahman et al. (2004), and Zamani-Nour et al. (2012) only sampled the oilseed subspecies (ssp. *oleifera*, ssp. *trilocularis*, and ssp. *dichotoma*), which prevented them from relating their results to the other *Brassica rapa* morphotypes and providing a full picture of the phylogenetic relationship of *B. rapa* subspecies. Tanhuanpää et al. (2016) showed brown and yellow sarson clustering together in STRUCTURE analysis, but did not use both subspecies in their Neighbor Joining dendrogram. Furthermore, they only sampled two accessions of ssp. *dichotoma* and one of their two accessions may have been misclassified as *B. rapa* ssp. *dichotoma* because it was named “yellow sarson.” The one remaining *dichotoma* line in the Tanhuanpää et al. (2016) study showed ~50% membership probability assignment for the yellow sarson subpopulation, with the second highest probability being for the *oleifera* subpopulation. With our improved sampling of ssp. *trilocularis* and ssp. *dichotoma* compared to previous studies combined with the other sampled subspecies, we were able to demonstrate both the relationship of ssp. *trilocularis* and *dichotoma* to other *B. rapa* subspecies as well as their relationship to each other.

Historically, the taxonomy of oilseed *B. rapa* has been complex. Taxa were frequently moved between *B. rapa* and *B. napus* in different classifications, were at times separated by seed color, and frequently revised for nomenclature (Gupta and Pratap, 2007). As such, the subspecies *dichotoma* may sometimes be used by breeders or seed banks to refer to both brown sarson and toria. Toria and brown sarson have different flowering time phenotypes, with toria flowering at least 2 weeks before brown sarson (Ahlawat, 2008). Variation in flowering time has been shown to contribute to phenological assortative mating in experimental populations of *B. rapa* (Weis and Kossler, 2004), suggesting population differentiation is possible between toria and brown sarson. Additionally, there are differing degrees of self-compatibility between toria and brown sarson. Toria has been found to be largely self-incompatible (McGrath and Quiros, 1992), while some varieties of brown sarson are self-compatible (Ahlawat, 2008). Our results potentially indicate that the polyphyly we see in ssp. *dichotoma* is caused by two genetically distinct varieties (toria and brown sarson) being grouped into a single subspecies. Toria and brown sarson have been previously recognized as different ecotypes within *Brassica campestris* ssp. *oleifera* (Hinata and Prakash, 1984), which potentially supports splitting them into different subspecies or as distinct varieties within the subspecies *dichotoma*. This hypothesis aligns with the concept of “overlumping” described in animal species by Funk and Omland (2003). Alternatively, the observed pattern may be due to hybridization between some ssp. *dichotoma* and ssp. *trilocularis* lines. Considering the heterozygosity rate for ssp. *dichotoma* lines that group with ssp. *trilocularis* is low (less than the population-wide average of 10%) and neither FastStructure nor PCA show signs of admixture this does not seem to be a likely explanation.

Finally, we highlight a potential for concern with the classification of the subspecies *oleifera*. Of the eight *oleifera* accessions in our analysis, three were found to be unknown *Brassica* polyploids, *one* clustered with *B. rapa* ssp. *chinensis*, and the Italian *oleifera* and Middle Eastern/South Asian *oleifera* accessions did not cluster together on the RAxML or FastStructure analyses. Because the accessions were found in multiple groups, and our initial sampling was nearly reduced by half due to species misidentification by USDA curators, as well as a lack of information about flowering time from GRIN-Global, it was unclear whether these issues were the result of *oleifera* being polyphyletic or were caused by a high incidence of misidentified accessions. In light of our results, variable assignment of *oleifera* to *B. napus* or *B. rapa* (Gupta and Pratap, 2007), and the frequently observed strong genetic differentiation between spring and winter *oleifera* accessions (Zhao et al., 2005, 2007, 2010; Del Carpio et al., 2011a,b; Tanhuanpää et al., 2016), the usage of *oleifera* as a subspecies seems inherently problematic.

### European and asian turnips

Additionally, our results suggest polyphyly for turnip accessions based on geography. Similar results have been found in previous studies (Zhao et al., 2005; Del Carpio et al., 2011a; Cheng et al., 2016a,b; Takahashi et al., 2016). However, our results provide the strongest support for the split between Japanese and European turnips (subpopulations 1, 2 in Figure 2). Takahashi et al. (2016), in addition to finding two groups of turnip (European and Japanese), also found strong admixture in accessions from continental Asia, especially those from Central/South Asia (broadly interpreted to include Pakistan, Afghanistan, and parts of India).

Our results shed further light on the relationship between Asian turnips and Central/South Asian accessions. Turnip-like accessions from Central/South Asia, specifically lines from Afghanistan, Pakistan, and India; oilseed lines such as *B. rapa* ssp. *dichotoma* (toria and brown sarson); and *B. rapa* ssp. *nipposinica* (mizuna) all grouped together forming a single population cluster, implying a further division between European turnips and continental Asian turnips/oilseed types. It has previously been established that mizuna has a common ancestor with oilseed rape, as opposed to the other leafy vegetable types that are thought to have been developed after the species entered China (Dixon, 2007). Likewise, Japanese turnips are thought to be derived from the Afghan variety of turnips that have smaller taproots and an ascending rosette (Dixon, 2007). Our FastStructure results seem to support these hypotheses concerning the genetic and evolutionary relationship of Central/South Asian and Japanese accessions.

Phylogenetic support for East Asian turnips originating from Central and South Asia was less clear as both Mediterranean (Italy), Middle Eastern/North African (Iraq and Egypt), and the Central/South Asian (Afghanistan, Pakistan, India) accessions did not form definitive clades as were found with European and Japanese turnips (Supplementary Figure 3). However, the general topology of the phylogeny did reinforce the FastStructure results as the Mediterranean, Middle Eastern, and Central/South Asian accessions were more closely related to the Japanese turnips than to the European turnips.

## Conclusions

In this study, we constructed a large SNP data set for 333 diverse, globally representative accessions of *B. rapa* assembled from the USDA NPGS in order to infer the population structure and phylogenetic relationship of the 10 sampled subspecies. Through our analyses, we identified a number of accessions that were misclassified as *B. rapa*, as well as several individuals that appear to have incorrect subspecies identification. These results can be used to aid and improve the curation of the *B. rapa* accessions curated by the NPGS. Additionally, our results indicated that both morphotype and geography contribute to the population structure of *B. rapa* subspecies. Using FastStructure, we identified major morphotype groups (turnip, oilseed, Chinese cabbage, and bok choy) and separated turnips by geographic origin (European vs. East Asian). We also found evidence for polyphyly in ssp. *dichotoma* and ssp. *oleifera* that can help facilitate a more genetically informed approach to taxonomic classification for these morphotypes. The strong population differentiation indicated by FastStructure and phylogenetic results was also reflected in the significant AMOVA and pairwise F_ST_ values observed in our analyses. These results emphasize the importance of morphotype on genetic diversity, but also indicated that there exists significant differences between the same morphotypes that are related to geographic origin. Geographic differences may have arisen through different domestication histories, introgression and backcrossing, or extensive geographic isolation. Future work in this area would greatly benefit from even more expanded sampling, including more turnips from both Europe, East Asia, and Central Asia; *Brassica rapa* ssp. *sylvestris* and other putative “wild accessions”; and more oilseed lines like ssp. *oleifera* and ssp. *dichotoma*. A population genetic analysis of this expanded sampling could help better inform us about the domestication history and potential convergence/introgression that was identified in this study. *One* potential approach would be to leverage a large number of genetic markers for a globally representative *B. rapa* diversity set to test hypotheses of introgression using population demographic models, such as ∂a∂I (Gutenkunst et al., 2009) and TreeMix (Pickrell and Pritchard, 2012). This data set and the results presented here can greatly facilitate these future investigations to further unravel the phylogenetic history and population dynamics of this valuable crop species.

## Author contributions

KB, EG, MG, JP, and JL prepared the manuscript. KB, HA, EG, and JL processed the sequencing data. KB performed bioinformatics and population genetic analyses. HA performed taxa quality control. LR and JL provided germplasm management and collection. KB, HA, LR, and JL managed experimental design and GBS data production. JP, MG, LR, and JL provided experimental design and coordination.

## Funding

This work is funded and supported by the USDA-ARS Crucifer Germplasm Committee Project No. 1910-21000-024-00D, USDA-NIFA/DOE Biomass Research and Development Initiative (BRDI) Proposal No. 2011-06476, NSF-IOS Award No. 1339156, and Cornell University startup funds.

### Conflict of interest statement

The authors declare that the research was conducted in the absence of any commercial or financial relationships that could be construed as a potential conflict of interest.
