# Supplementary material for: Population Structure and Phylogenetic Relationships in a Diverse Panel of Brassica rapa L
Source: Front Plant Sci. 2017 Mar 13;8:321. doi: 10.3389/fpls.2017.00321 (PMC5346582; doi:10.3389/fpls.2017.00321)
Supplement: Supplementary file 3 [file DataSheet1.DOCX]

| Step in GBS pipeline | Key arguments | | Results | |
| --- | --- | --- | --- | --- |
| FastqToTagCountPlugin | -e ApeKI  -s 400,000,000 | Enzyme used to create GBS library  maximum number of reads allowed | C6NNTANXX_1.cnt  C6NNTANXX_2.cnt  C6NNTANXX_3.cnt  C6NNTANXX_4.cnt | Reads: 233530205  w/barcode&cut site:  216247328  tags: 12649588  Reads: 207284306  w/barcode&cut site:  193412012  tags: 11078943  Reads: 182328878  w/barcode&cut site:  171520659  tags: 14831568  reads:200810967  w/barcode& cut site: 188031347  tags: 12281901 |
| MergeMultipleTagCountPlugin | -c 5  -t | Tags must appear at least 5 times across 386 barcoded samples to be output  Maximum tag number in merged TagCount file  Output as Fastq | tagsRead=50773385 outCnt=3025369  Output : BrapaMasterTags.cnt.fq | |
|  |  |  |  | |
|  |  |  |  | |
| SAMConverterPlugin |  | Align using BWA -aln | Topm file w/  1123261 were aligned to unique positions  160867 were aligned to multiple positions  1741241 could not be aligned  42% aligned to reference | |
| FastqToTBTPlugin | -e ApeKI  -o tbt  -y |  |  | |
| -MergeTagsByTaxaFilesPlugin |  |  |  | |
| DiscoverySNPCallerPlugin | -mnF -2.0 -mnMAF 0.02 -mnMAC 10000  minTaxaW/Locus:38  - includeRare:false includeGaps:false |  | 277,247 SNPs | |
| MergeDuplicateSNPsPlugin | -misMat 2.0  -callHets | Call only biallelic loci | 253,332 SNP | |
| VCF | -remove-indels  -max-allele=2  -call-hets |  | 253,332 SNPs | |
| FilterClustersScript | -cls 3 | Removes cluster with 3 or more SNPs | 226,619 SNPs | |
|  |  |  |  | |
|  |  |  |  | |
|  |  |  |  | |
|  |  |  |  | |
|  |  |  |  | |
|  |  |  |  | |
|  |  |  |  | |
| VCF | --max-mean-depth=140 |  | 226,306 SNPs | |
| FilterHetzScript | -htz_threshold = 0.4  -htz >=min(hmz1,hmz2) | removes SNPs with heterozygosity over 40% and sites with heterozygosity rate over two time the minor allele frequency | 55,610 SNPs | |
| TASSEL5 GUI | MnSCov 307  MnTCov 0.1 | Remove sites not present in at least 307 taxa and remove taxa that have fewer than 10% of all sites | 18,272 SNPs | |
|  |  |  |  | |
|  |  |  |  | |
