# Supplementary material for: Population Structure and Phylogenetic Relationships in a Diverse Panel of Brassica rapa L
Source: Front Plant Sci. 2017 Mar 13;8:321. doi: 10.3389/fpls.2017.00321 (PMC5346582; doi:10.3389/fpls.2017.00321)
Supplement: Supplementary file 4 [file DataSheet2.DOCX]

Brassica rapa ssp.chinensis

Baby bok choy by Rusty Clark

URL=

(<https://www.flickr.com/photos/rusty_clark/8467755927/in/photolist-dUgsPR-8RCT22-dW1diq-2jjTF2-D1zqAw-kGzGZ-6mWVqF-6vaXgx-7rrdLs-6aqdfX-4cn5rM-6HLBxv-aQfQHP-ffDk5F-5T8SuZ-6CM7fQ-bqSp1B-8CjLs2-9mywC9-cMSNCo-dMmgUF-5243ov-drNGQe-6dpXwF-7YMt6M-726v3d-8awTVk-bbU3Qk-7Nssqv-LWsCG-6pJHMQ-je5PB-4jSskd-7DQKAd-a3R7ds-bdgEQa-dUn5a9-9GHxP8-dk8xgn-Fdj8zT-86bSq2-bkKsM1-9pEsy1-qmwtNX-jStfwk-7LDM6u-eX78Wm-efz3iw-je5WD-brK3Lq>)

Creative commons (<https://creativecommons.org/licenses/by-nc/2.0/>)

Brassica rapa ssp. oleifera:

Brassica rapa 3, by J_Arlecchino URL=(<https://www.flickr.com/photos/116797173@N07/16332796664/in/photolist-qTgRqu-DAFVrm-cpVx8E-DuksCM-cpVzAo-cpVzX1-GHWgUr-DMeHux-cpVzkQ-D65uz5-GiRAYY-GN6GD4-D6pAiF-cpVyoJ-D1Y6jd-D65txf-DUb741-cpVxr9-DQ3a9e-DSnFXj-Dq5C18-cpVwPh-DViEAL-cpVxMU-D1M9JG-cpVy6q-D5S94u-D2b13r-DqcN5x-DYFZXZ-553HUL-cpVz4S-DKvM5M-cpVyBu-DYG2rF-DVubPk-DKvKzH-cpVAdW-DNgoWb-EatjLG-D7kXac-6a8CLt-EgFfd3-DUy3Gc-E8ycNw-Dp9jJH-DbMrMQ-D1E6Pr-DKvLzD-DQrAXT>)

Creative commons(<https://creativecommons.org/licenses/by-nc/2.0/>)

Yellow sarson:

Yellow mustard seeds, by Jessica and Lon Binder

URL=(<https://www.flickr.com/photos/lonbinder/3468485392/in/photolist-6hqJ14-6huTSh-AeKqP-7QXrby-3CX7Fb-9dKCy2-bbsGb2-9wFLLm-7Lcr4g-pth9ym-EnySed-J33VP-eB6ygX-kBiaFm-HjWvv-bbr1X8-dWBmfn-76dykU-sAMGY-8DU2fb-8MGN3R-2my8mW-6fV1VZ-7fuAiU-7yxbkM-keR6qe-2roFgh-onhNvy-6prPhA-bg18RF-f1HNZh-f1txZK-8T9HtJ-d3bpQ-on2Z3e-f1tANZ-673sVY-94W9wd-gc2it2-8496rX-s8ZZbj-nkGquR-6fV1W2-677nh1-gc1JJf-9AnC27-RruDU-3Xf73a-6pUkCC-revKcw/>)

Creative commons (<https://creativecommons.org/licenses/by-nc-nd/2.0/>)

Brown sarson:

Week_18_2009_05 by four4dots

URL=(<https://www.flickr.com/photos/four4dots/4214289776/>)

Creative Commons (<https://creativecommons.org/licenses/by-nd/2.0/> )

Brassica rapa Ssp. pekinensis:

Baechu Kimchi: Napa aka Chinese Cabbage by I Believe I can Fry

URL=(<https://www.flickr.com/photos/johnnystiletto/5812750434/>)

Creative Commons(<https://creativecommons.org/licenses/by-nc/2.0/> )

Turnip:

Suzuna Kabu Turnip by GetHiroshima.com

URL=(<https://www.flickr.com/photos/gethiroshima/11826827715/in/photolist-j26zQF-nHNM4Q-D8oRhP-hJZKrF-dg5XYS-3L6oMH-dSMEhi-DRNRT-sQMnPM-633S26-sybpCu-gZXwBW-7RB9RZ-8H3EGX-7BU92r-sQMpSK-sybrxS-acww4U-8uKufs-dsYtJm-5HoHbE-pH6BPm-rEknD7-8V7XoZ-oZJYiC-bzs978-5HPDeb-sNsfad-5F6W9h-dAja4E-623jF-rEkkxU-7SarWa-XhNxu-bo9peq-BknogM-edv1eU-8g8jm4-cApnpb-wy7b5-pH6EM5-GBYWUT-8jEbmP-7fn3ca-jw2waX-b27F6z-6eHrEn-9UpujT-bVfVHq-6mPijL>)

Creating Commons <https://creativecommons.org/licenses/by-nc/2.0/>
