# Supplementary material for: Population Structure and Phylogenetic Relationships in a Diverse Panel of Brassica rapa L
Source: Front Plant Sci. 2017 Mar 13;8:321. doi: 10.3389/fpls.2017.00321 (PMC5346582; doi:10.3389/fpls.2017.00321)
Supplement: Supplementary file 5 [file DataSheet3.DOCX]

Supp. Figure 1. Results of ChooseK script showing marginal likelihood for each sampled K value in FastStructure

Supp. Figure 2. Full FastStructure results at K=6 with USDA PI numbers listed for each sample. Color coding corresponds to Figure 2

Supp. Figure 3. Full RAxML phylogeny with tips labeled with USDA PI numbers and nodes labeled with bootstrap values. Color coding corresponds to Figure 2

Supp Table 1. Full list of all 364 accessions sampled. Geographic origin, subspecies identification, designated subpopulation cluster, and information about missing data and observed heterozygosity for each accession are listed. Duplicate plant samples used for quality control of genotyping are listed on in the duplicate samples sheet

Supp Table 2. List of accessions discarded after taxa quality filtering based on flow cytometry. Accessions listed without flow cytometry results were found to cluster with the putative *B. napus* lines in a preliminary FastStructure analysis at K=2

Supp File 1. Steps, commands, and outputs of GBS v1 pipeline implemented in TASSEL v 4
