# Supplementary figures and images for: Population Structure and Phylogenetic Relationships in a Diverse Panel of Brassica rapa L
Source: Front Plant Sci. 2017 Mar 13;8:321. doi: 10.3389/fpls.2017.00321 (PMC5346582; doi:10.3389/fpls.2017.00321)

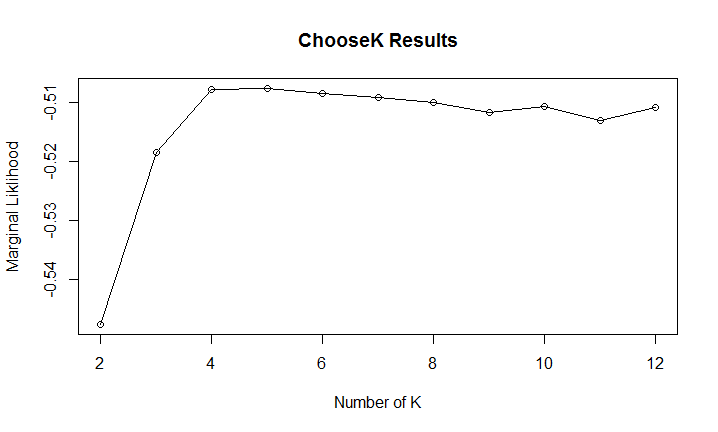

Supplement: Supplementary file 6 [file Image1.PNG]

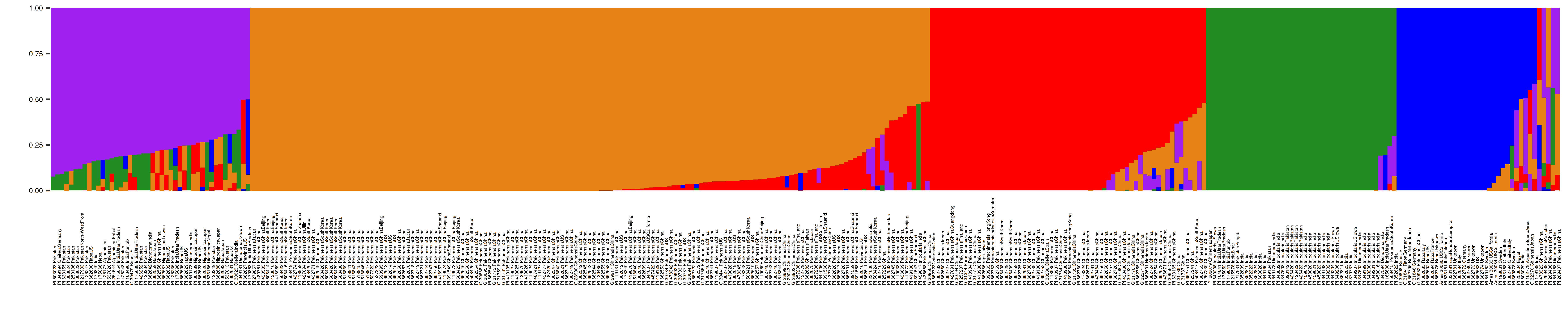

Supplement: Supplementary file 7 [file Image2.JPEG]

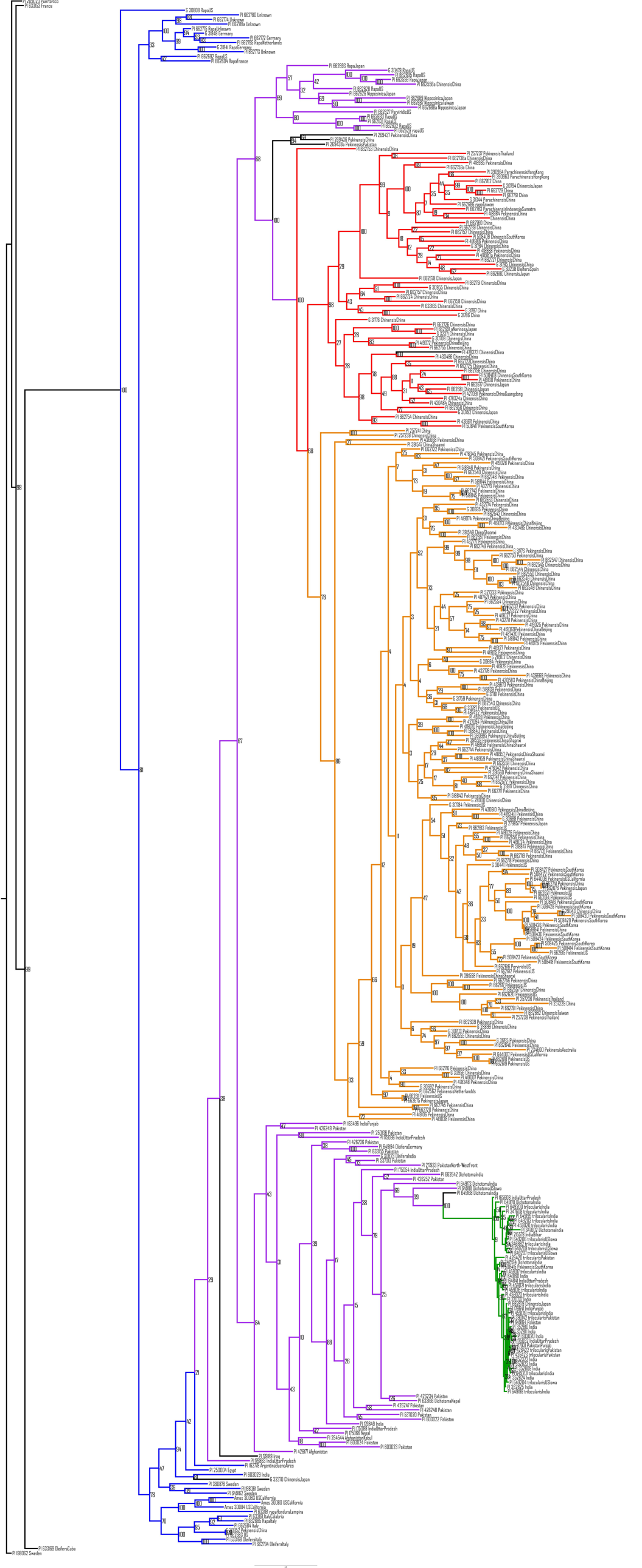

Supplement: Supplementary file 8 [file Image3.JPEG]
